# Supplementary material for: Statistical modeling of adaptive neural networks explains co-existence of avalanches and oscillations in resting human brain
Source: Nat Comput Sci. 2023 Mar 20;3(3):254–63. doi: 10.1038/s43588-023-00410-9 (PMC10766559; doi:10.1038/s43588-023-00410-9)
Supplement: Supplementary file 1 — Supplementary Methods, Table 1, Figs. 1–26 and Kullback–Leibler divergence analysis. [file 43588_2023_410_MOESM1_ESM.pdf]

nature computational science

Article

https://doi.org/10.1038/s43588-023-00410-9

Statistical modeling of adaptive neural networks explains co-existence of avalanches and oscillations in resting human brain

In the format provided by the authors and unedited

Contents

1 Supplementary Section-1. Supplementary Methods 1
1.1 Entropy production 1
1.2 Adaptive Ising model with local feedback 2
1.3 Controlling average activity level in the model 4
1.4 Mapping between the adaptive Ising model and an E-I network 5
1.5 The role of topology 6
1.6 Parameter fitting 7
1.7 Alternative approaches to parameter inference 8
1.8 Surrogate data and statistical analysis 11

2 Supplementary Section-2. Kullback–Leibler divergence analysis 13

3 Supplementary Section-3. Supplementary figures 14

1 Supplementary Section-1. Supplementary Methods

1.1 Entropy production

The model has a clearly identified Hamiltonian and free energy, and the entropy production can be assessed with tools of stochastic thermodynamics [3]. We have that the instantaneous rate of "work" made by the system is ($M = mN$)

$$\dot{w} = -M\frac{dh}{dt} \tag{1}$$

[4]. Assuming that, on average, the system is stationary, the entropy rate will be (brackets stand both for averages in time and over ensembles)

$$\langle \dot{S} \rangle = -\left\langle M\frac{dh}{dt} \right\rangle. \tag{2}$$

In our model this will be

$$\langle \dot{S} \rangle = c\langle m^2 \rangle N. \tag{3}$$

Essentially, the entropy production coincides with the "order parameter", and it becomes extensive above the critical point, i.e, for $\beta > \beta_c = 1$,

$$\langle \dot{S} \rangle = c(\beta - 1)N. \tag{4}$$

In the regime characteristic of our MEG signals, $\beta < \beta_c = 1$, the entropy production is sub-extensive (with a singularity at β_c, that is eventually rounded-off

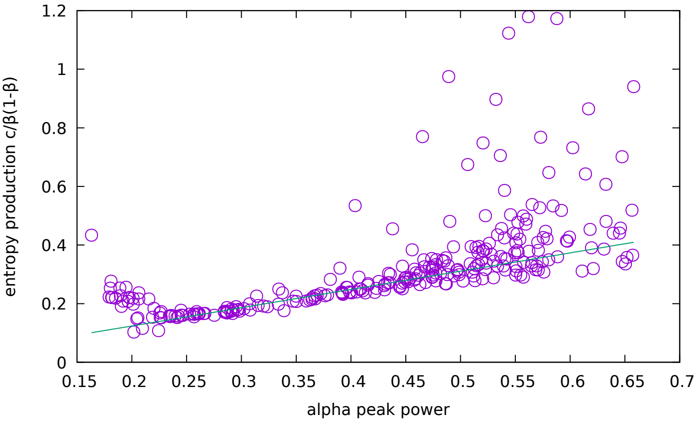

Supplementary Figure 1 Analysis of the entropy production as a function of the power in the alpha band of MEG signals. Entropy production increases linearly with the power in the alpha band (green thick line = linear model fit $y = ax + b$).

taking into account finite size effect), namely

$$\langle \dot{S} \rangle = \frac{c}{\beta(1-\beta)}. \tag{5}$$

For $c = 0$ the static Ising model is recovered, and, correctly, the entropy production rate becomes zero. In Supplementary Figure 1 we show the scatter plot of the inferred entropy production rate for the experimental signals versus the power in the alpha band. We observe a clear correlation between the two quantities, with the entropy production increasing linearly with the power in the alpha band (green thick line = linear fit). This suggests that an increase in entropy production in the brain could signal the approach to a dynamical bifurcation in the phase space.

Recently, entropy production in the brain has been studied with an asymmetric Sherrington-Kirkpatrick (SK) model, and associated with local asymmetric interactions [5]. However, entropy production could also be related to out-of-equilibrium phase transitions between different regimes, as, for instance, in the symmetric SK model. On the other hand, the adaptive Ising class is inherently out-of-equilibrium and analytically tractable, with a clear phase diagram whose bifurcation lines can be analytically connected with entropy production behavior, as shown above.

1.2 Adaptive Ising model with local feedback

In the simple mean field setting that we consider for our adaptive Ising model, the extension from global to local fields shall not affect the overall dynamical behavior with regard to average quantities, as we show in the following.

Consider a fully connected model with local fields, h_i, whose Hamiltonian is

$$H = -\frac{J}{N} \sum_{i<j} s_i s_j - \sum_i h_i s_i \tag{6}$$

The partition function can be calculated by neglecting second order terms (Curie-Weiss approximation, where in the quadratic term in the Hamiltonian above we approximate $s_j \sim \langle s \rangle$):

$$Z = \sum_{\mathbf{s}} e^{-\beta H} \sim \sum_{\mathbf{s}} \prod_i e^{\beta(J\langle s \rangle + h_i)s_i} = \prod_i 2\cosh(\beta(J\langle s \rangle + h_i)) \tag{7}$$

By including the feedback and applying linear response to $m_i = \langle s_i \rangle$, one has the system of dynamical differential equations (using $m = \langle s \rangle = \frac{1}{N}\sum_i \langle s_i \rangle$ and $J = 1$):

$$\begin{aligned} \dot{m}_i &= -m_i + \tanh(\beta(m + h_i)) \\ \dot{h}_i &= -c m_i \end{aligned} \tag{8}$$

If one sets $m_i = m$ for each i, this system has the same solution(s) as the homogeneous system under the influence of a global field. In what follows, we indicate the global magnetization and field as $m_g(t)$ and $h_g(t)$, respectively. We can test the stability of these solutions by considering m_i and h_i as small perturbations to the global fields, namely $m_i = m_g + \delta_i$, $h_i = h_g + \delta_{hi}$. By substituting such expressions into the Eqs. 9 and using $\tanh(a+b) = (\tanh a + \tanh b)/(1 + \tanh a \cdot \tanh b)$ and neglecting higher order terms, we obtain the linearized system

$$\begin{aligned} \dot{\delta}_i &= -\delta_i + \beta\delta_{hi}(1 - \beta\tanh^2(\beta(m_g + h_g))) \\ \dot{\delta}_{hi} &= -c\delta_i \end{aligned} \tag{9}$$

If $\beta < \beta_c$, $m_g = h_g = 0$, the eigenvalues of the Jacobian

$$\lambda_\pm = \frac{-1 \pm \sqrt{1 - 4\beta c}}{2} \tag{10}$$

have negative real part, i.e. perturbations get exponentially suppressed. This is confirmed by numerical simulations of the system Eq. 10 as illustrated in Supplementary Figure 2.

Taken together, this mean field approximation suggests that the system with local feedback retains qualitatively the same features as for the global feedback case. A full assessment of the impact of local fields, beyond simple mean field approximations, would require further in-depth investigations.

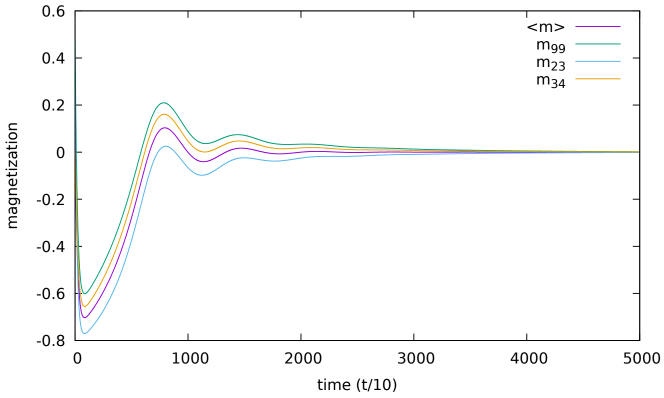

Supplementary Figure 2 Magnetization for the model with local feedback. Magnetization as a function of time from numerical simulations of the system of ODEs (9) for three different spins and for $c = 0.01$, $\beta = 0.95$ and $N = 100$ variables. The global magnetization, m_g, is shown in purple. Initial conditions are random in $(-1, 1)$. The initial perturbations applied to the different spins all eventually decay, and all m_i converge to the same value $m = \langle m \rangle = 0$.

1.3 Controlling average activity level in the model

The model can be modified to modulate the average level of neural activity by inserting a constant feedback that fixes it to some desired value $m_0 \neq 0$. An $m_0 \neq 0$ would be needed, for instance, in case of a change of variables $s_i \to \sigma_i$ ($\sigma_i = 0, 1$) to compensate for the associated shift in the level of neural activity.

The ODE system for the model with a bias $m_0 \neq 0$ on the magnetization is

$$\dot{m} = -m + \tanh(\beta(m + h)) \tag{11}$$

$$\dot{h} = -c(m - m_0) \tag{12}$$

The stationary point will be then

$$m_s = m_0 \tag{13}$$

$$h_s = h_0 = \frac{\operatorname{atanh}(m_0)}{\beta} - m_0 \tag{14}$$

A linear perturbation analysis shows that the critical point will be now at

$$\beta_c = \frac{1}{1 - m_0^2}. \tag{15}$$

This is once again an Andronov-Hopf bifurcation line where the real part of a pair of complex eigenvalues changes sign, indicating the emergence of a limit cycle. The bifurcation line is preceded by a resonant regime as in the case for $m_0 = 0$. Beyond the critical point, an approximate analytical solution can be worked out for $\beta >\sim \beta_c$ (harmonic oscillations) by a two-time expansion

(Strogatz 2018). If we set $\epsilon = \beta - \beta_c$ we have

$$m - m_0 \sim \sqrt{\epsilon} \cos\left((1 + \frac{1}{2}\epsilon)\sqrt{c}t + \phi_0\right), \tag{16}$$

which gives an oscillating magnetization as in the case $m_0 = 0$. This shows that our framework can accommodate for a shift in global activity levels—or a reparametrization of spin variables s_i from $(-1, +1)$ to $(0, 1)$—and the qualitative behavior of the model is preserved, at least in the simple mean field setting.

1.4 Mapping between the adaptive Ising model and an E-I network

We first encode a classic E-I model that leads to sustained oscillations, into an Ising model framework. We can think of this model as a single network of N units whose coupling matrix J_{ij} is asymmetric and is structured into two blocks that correspond to an excitatory and an inhibitory subpopulation. Specificcally, the network consists of a population 1, which is self-exciting with strength J_{11} and which excites population 2 with strength J_{12}, while population 2 is inhibiting population 1 with strength J_{21}.

It can be demonstrated that the mean-field dynamics of this stochastic system of Ising-like neurons in the limit of large populations is described by a Liouville deterministic equation of the form (m_i $i = 1, 2$ is the average spiking rate of population i) [6]:

$$\dot{m}_1 = -m_1 + \tanh(J_{11}m_1 + J_{21}m_2) \tag{17}$$

$$\dot{m}_2 = -m_2 + \tanh(J_{12}m_1) \tag{18}$$

$$\tag{19}$$

The E-I network has an ergodic state where ($m_1 = m_2 = 0$). Stability analysis to small perturbations of this state reveals an Andronov-Hopf bifurcation towards self-oscillations, when $J_{11} = 2$ and $-J_{12}J_{21} > 1$. Upon matching the coefficients of such a linear expansion

$$\ddot{m} + (1 - \beta)\dot{m} + c\beta m = 0 \tag{20}$$

$$\ddot{m}_1 + (2 - J_{11})\dot{m}_1 + (1 - J_{11} - J_{12}J_{21})m_1 = 0 \tag{21}$$

we get an approximate mapping into the parameters β, c of the simplest adaptive Ising model:

$$J_{11} = \beta + 1 \tag{22}$$

$$J_{12} = \sqrt{\beta(1 + c)} \tag{23}$$

$$J_{21} = -\sqrt{\beta(1 + c)}. \tag{24}$$

1.5 The role of topology

Phenomenological arguments, supported by preliminary results, suggest that adding the feedback that drives the adaptive Ising model out of equilibrium does not change the fundamental nature (e.g., critical exponents) of the critical point. This permits us to directly translate existing equilibrium results—including the Landau-Ginzburg theory and the renormalization group—into the non-equilibrium setup to model cell types (e.g., inhibitory vs excitatory) or capture empirically established topological features of real neural networks: an example would be the introduction of scale-free topology among excitatory neurons into our model, as shown in the following.

One of the most interesting questions about synchronization in neural networks is how general features of the interaction topology affect the collective behavior, i.e., how structure affects function in general terms [7]. From a modeling perspective, most efforts have focused on studying the Kuramoto model (KM) [8], where the individual excitable units are already postulated to be oscillators (for a discussion about this point see [9]). Nevertheless, no exact analytical results for the KM on general networks are available up to now, with an intense debate currently focusing on the nature of the onset of synchronization in strongly heterogeneous topologies [10].

In contrast, we provide here an heuristic argument that the adaptive Ising model directly inherits the wealth of knowledge accumulated about its equilibrium counterpart, in particular, with regard to the features and the location of its critical point(s). The critical point characteristics have been *rigorously* determined in several geometries, from dimensional lattices to complex networks and small world [11–13].

The fact that equilibrium Ising results can be generalized to the adaptive case can be seen directly from the application of the linear response theory, upon considering the Landau expression for the free energy: by construction, the bifurcation point of the dynamical model coincides with the critical point of the underlying equilibrium model.

For instance, for the case of uncorrelated tree-like random graphs, described by a degree distribution $P(k)$ [14], the linear response applied to a Curie-Weiss approximate expression for the free energy [15] leads to the following approximate dynamical equations for the firing rates of nodes with degree k, m_k (where $\langle k\rangle$ is the mean degree, $\langle m\rangle = \sum_k P(k) m_k$ is the average firing rate and $\langle m_v\rangle = \sum_k \frac{k}{\langle k\rangle} P(k) m_k$ is the average firing rate upon following a random link):

$$\dot{m}_k = -m_k + \tanh(\beta(k\langle m_v\rangle + h)) \quad \forall k \tag{25}$$

$$\dot{h} = -c\langle m\rangle. \tag{26}$$

As it can be easily verified by linearizing around the stationary solution $m_k = h = 0$, these equations show that the model has a bifurcation point located at the same position as the equilibrium critical point, i.e. $(\beta J)_c = \frac{\langle k^2\rangle}{\langle k\rangle}$

(a more refined calculation [15] based on asymptotically exact Bethe-Peierls approximation gives $(\beta J)_c = -\frac{1}{2}\log(1 - 2\frac{\langle k \rangle}{\langle k^2 \rangle}))$.

This simple example shows that the inverse temperature gets renormalized by the branching ratio [14] $\frac{\langle k^2 \rangle}{\langle k \rangle}$, a topological measure of the density of links, or synaptic connections in our context, that could be considered itself as the key control parameter driving the system in and/or out the synchronized phase. A direct consequence for our case is that if the topology we were considering were scale free, i.e. with an heavy tail for the degree distribution $P(k) \sim k^{-\gamma}$ $\gamma < 3$, then $\beta_c \to 0$ and the system would always be in the synchronized phase, a feature shared by many collective phenomena in strongly heterogeneous networks [13]. In our case, subcritical dynamics is inferred from data and the scale-free topology is not appropriate, but the reasoning here demonstrates clearly how known facts about equilibrium Ising on different topologies can directly translate into the insights of the adaptive Ising model.

Preliminary model simulations show that the exponent ζ, which connects avalanche sizes and durations, is affected by the connectivity and, furthermore, more closely matches the value of ~ 1.3 characteristic of the data for nearest-neighbor 2D connectivity on a square lattice. This suggest that the exponent ζ could be influenced by the underlying network connectivity. Regarding this point, recent numerical work showed that one can obtain $\zeta \simeq 1.3$ by subsampling the activity of models that are otherwise constructed to have $\zeta = 2$ [16], which suggests that subsampling in brain activity recordings could alternatively explain $\zeta \simeq 1.3$, the value reported here and in other studies [17].

1.6 Parameter fitting

The parameters β and c where chosen as the minimizers of L_2 error $D(c, \beta)$ between the discrete autocorrelation function estimated from data and the approximate analytical expression of the latter in the model, i.e.

$$D(c, \beta) = \frac{1}{T}\sum_{t=1}^{T}(C_{exp}(t) - C_{mod,\beta,c}(t))^2 \tag{27}$$

where

$$C_{mod,\beta,c}(t) = e^{-\gamma t}(\cos(\omega t) + \frac{\gamma \sin(\omega t)}{\omega}) \quad \beta = 1 - 2\gamma \quad c = \frac{\gamma^2 - \omega^2}{4(1-\gamma)} \tag{28}$$

Autocorrelation function is estimated from the sensor data (z-scored by its mean and variance $x(t) \to \frac{x(t)-\bar{x}}{\sigma}$ to get a zero-mean unit variance signal):

$$C_{exp}(\tau) = \frac{1}{N-\tau}\sum_{t=0}^{N-\tau} x_t x_{t+\tau}, \tag{29}$$

which is a standard (maximum-likelihood) estimator for the autocorrelation assuming IID gaussian noise. We typically use $N = O(10^5)$ samples, sufficient for this procedure to converge to estimates with very small empirical error; our standard error for $C(\tau)$ is of the order 10^{-3}, estimated via bootstrap. The function $D(\beta, c)$ is an analytic function of two variables whose minima can be found with elementary methods, in our case, gradient descent.

In our analysis we set $J = 1$ and $\beta_c = 1$, and thus what we infer in essence is

$$\frac{\beta J}{(\beta J)_c}. \tag{30}$$

The parameter β can be connected with the typical timescales of the system analytically through the result of the linear stability analysis (see main text Eq. 3). The relaxation/decorrelation time of the system is

$$\gamma = \frac{2}{1-\beta}. \tag{31}$$

The β values inferred from MEG data are clustered below the critical point $\beta_c = 1$, i.e. $\beta = 0.986 \pm 0.006$ (see Supplementary Figure 8). An interesting point that we aim to address in future work is how close such values are to the critical point. While using Eq. 31 one could compare the inferred β values with the typical timescales of the the system, proper definition of “closeness to criticality” would require defining “inherent” metrics in phase diagrams able to take into account possible inference biases [23]. In essence given a stochastic trajectory $m(t)$, if one has the expression of the probability conditioned to parameters, $P(m(t)|\vec{u} = (\beta, c))$, this metric is given by the so-called Fisher information matrix

$$g_{ij} = \left\langle \frac{\partial^2 \log P}{\partial u_i \partial u_j} \right\rangle, \tag{32}$$

where the average is performed over stochastic trajectories. Such evaluation could potentially be performed with the field theory tools used in [24].

1.7 Alternative approaches to parameter inference

The procedure to fit β and c makes use of the analytical form of the autocorrelation $C(t)$, which is only valid for $\beta < \beta_c$. To validate our approach and further verify that the data can be described by the Langevin equations of the model (Eq. 1, main text), we compare it with a more general inference method recently developed by Ferretti et al [25].

We performed the comparison between the Ferretti method (FM) and our autocorrelation matching (AM) using multiple chunks of $N = 1000$ data points each. This choice was only dictated by machine time constraints. Indeed, the machine time for the FM algorithm grows as N^3 (Supplementary Figure 3).

We first tested FM on time series generated by simulating our model at different values of β and c. We found that the parameters inferred via FM are in good agreement with those obtained via AM, and with the underlying ground

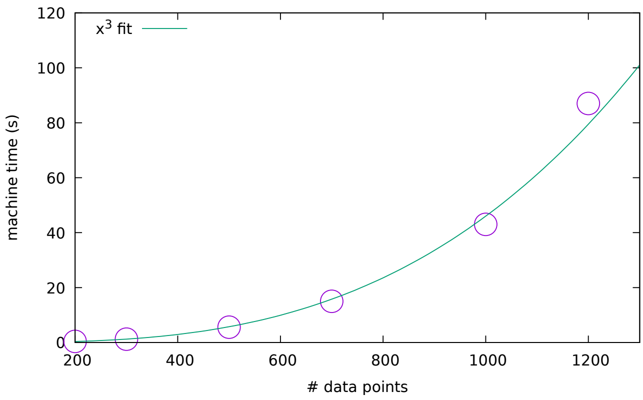

Supplementary Figure 3 Performance of the Ferretti method. Machine time versus the number of data points N for the Ferretti method (FM) [25]. The machine time grows approximately as N^3 (green line = cubic polynomial fit).

truth (Supplementary Figure 4). We note that, for the inference with the FM, we had to use the time series $h(t)$, i.e. the integral of the magnetization $m(t)$, which is much smoother than $m(t)$. For $m(t)$ we found that the algorithm does not converge, suggesting that FM is sensitive to the amount of noise present in the time series.

Given the fragility of the FM algorithm with respect to noise, we wondered whether it can estimate parameters well from empirical data. We compared the FM inference from $h(t)$ of a synthetic signal with the $h(t)$ of a real signal with the same length and exactly matching autocorrelation function (and thus identical β as identified by AM). Indeed, FM produced significantly different estimates of β for the real and synthetic signals. To verify that these issues are not due to the limited number of data points used to perform the inference with FM, we further tested FM on synthetic signals with different noise levels obtained from a linear combination between $h(t)$ and $m(t)$, namely $s(t) = h(t) + bm(t)$ with $0 < b < 1$. By construction, such signals have a fixed β, c, and an autocorrelation that is invariant of b. We found that, already for b as small as 0.1, the FM underestimates β (Supplementary Figure 5); the bias of the FM method thus depends on b. For larger b values, the method did not converge. This suggests that noise plays a key role in the FM performance. In contrast, AM identifies the parameters robustly (by construction) for any value of b. Despite our attempts, on our data, FM can thus not provide a reliable benchmark against which to compare and calibrate the performance of our autocorrelation-matching (AM) approach.

Lastly, we compared FM and AM on empirical MEG signals in greater detail. For the parameter c, we found a substantial agreement between the two methods, but the inferred errors of the FM were large due to the limit on timeseries length. As for β, we found that both methods consistently place the system slightly below the critical point $\beta_c = 1$ (Supplementary Figure 6),

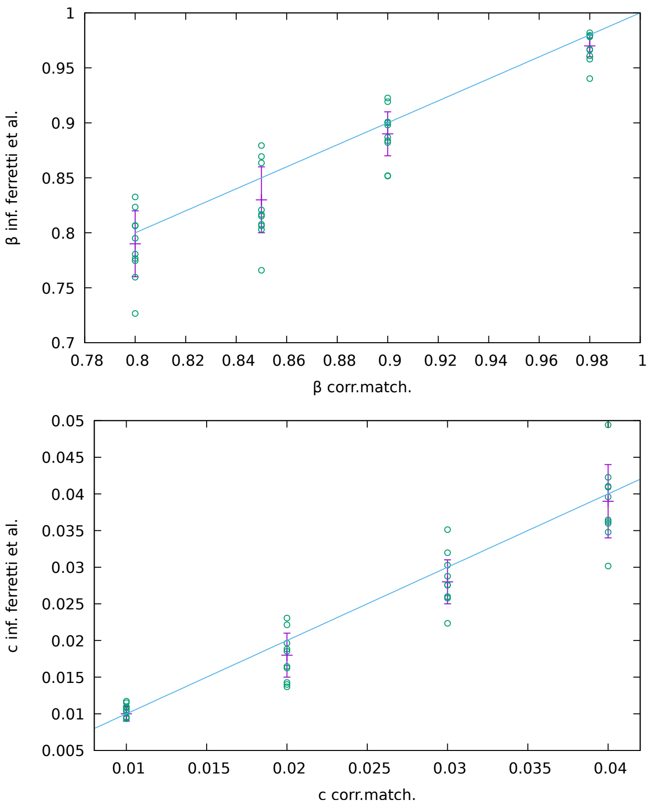

Supplementary Figure 4 Comparison between the Ferretti method and the autocorrelation matching on data from model simulations. Inferred β (upper plot) and c (lower plot) from the Ferretti method (FM; y axis) and the autocorrelation matching (AM; x axis) in synthetic data generated from model simulations. The parameters inferred via FM are in good agreement with those obtained via AM, and with the underlying ground truth. We note that AM recovers the ground truth by construction. We considered time traces simulated from the model in four different conditions $\beta = 0.8, 0.85, 0.9, 0.98$ and $c = 0.01, 0.02, 0.03, 0.04$, respectively. The cyan tick line represents the curve $y = x$. Error bars indicate the standard deviation ($n = 10$ synthetic data chunks of 1000 points each).

but with significant biases likely due to the application of the FM method to noisy empirical data, as explained above.

Overall these results indicate that, for controlled signals with low noise, FM and AM provide results that are in quantitative agreement with each other and with the ground truth (Supplementary Figure 4). This shows that our parameter estimates via autocorrelation are consistent with an inference method that does not assume a specific form of the autocorrelation, when that method is applicable.

Although not exhaustive, our analysis further indicates that observed biases in FM do not come from using a limited number of data points ($N = 1000$), but rather from the noise present in the real signals. Indeed, the FM shows a

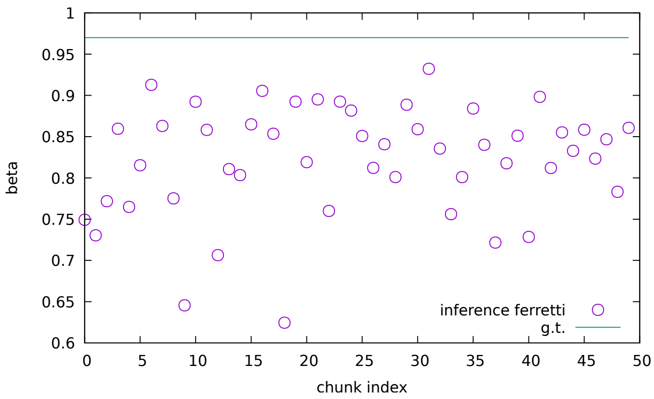

Supplementary Figure 5 Performance of the Ferretti method on model signals with added noise. Inferred β from the Ferretti method across chunks of a synthetic time series $s(t) = h(t) + 0.1m(t)$ obtained combining h and m from a model simulation with $\beta = 0.97$. Each chunk contains 1000 data points. FM is always below the ground truth (cyan tick line), and thus systematically underestimates β.

general instability even on synthetic data if the noise is too large. This plus other non-linear/non-gaussian properties of the MEG data may be responsible for the discrepancy observed in real data between the two methods. These difficulties, as well as the computational complexity of the FM, lead us to conclude that AM (subject to more a priori restrictions on the signal class) is nevertheless preferable for our application to the FM (which is theoretically more powerful and generic, but seems to suffer from technical limitations).

Finally, we note that we implemented the Ferretti algorithm to fit a linear model, as shown in [25]. To probe the dynamics beyond linear terms, ongoing work is focused on the recent inference method developed by Brückner et al [26].

1.8 Surrogate data and statistical analysis

Surrogate data

Surrogate signals are obtained by random phase shuffling of the original continuous MEG signals. A Fourier transform of each sensor signal is performed, the corresponding phases are randomized while amplitudes are preserved. The surrogate signals are then obtained by performing an inverse Fourier transform. The random phase shuffling destroys phase synchronization across cortical sites while preserving the linear properties of the original signals, such as power spectral density and two-point correlations [27].

Statistical analysis

The reported $p-$value for the relationship between β and the power in the alpha band in Figure 2D and β and α in Figure 2F rejects the null hypothesis

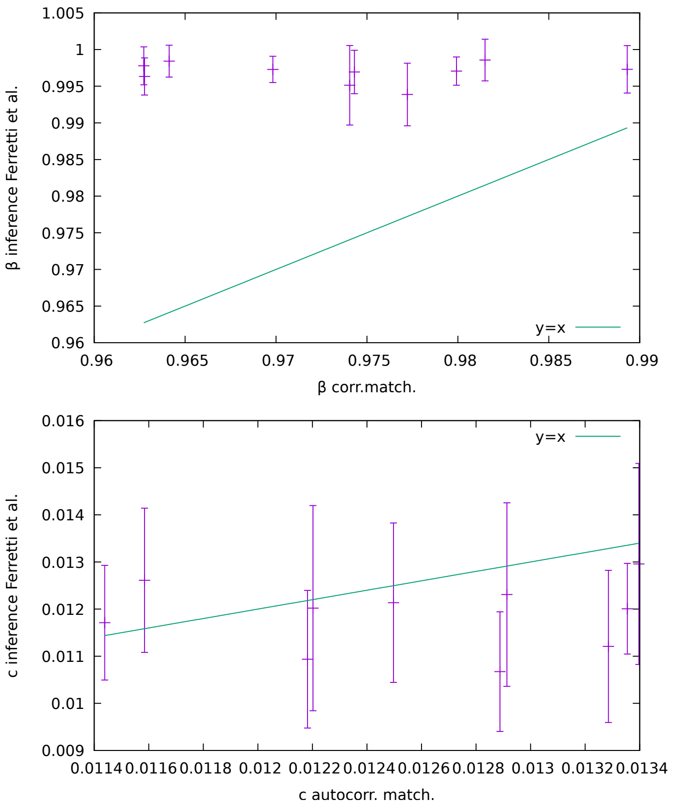

Supplementary Figure 6 Comparison between the Ferretti method and the autocorrelation matching for the inference of model parameters from MEG signals. Inferred β (top) and c (down) from the Ferretti method (FM; y axis) and the autocorrelation matching (AM; x axis) for $n = 10$ MEG signals in the α band from a single subject. Error bars indicate the standard deviation. For β (top), we observe that both methods consistently place the system slightly below the critical point $\beta_c = 1$, but with significant biases likely due to the application of the FM method to noisy empirical data. For the parameter c (bottom), we find a substantial agreement between the two methods, although the inferred errors of the FM are large due to limitation in time series length (see text).

that $y =$const (in other words, the p-value is the significance of the nonzero linear regression coefficient).

2 Supplementary Section-2. Kullback–Leibler divergence analysis

To quantify how close a model distribution $P_{\rm m}(X)$ is to the data-derived average distribution $P_{\rm d}(X)$, where X can be the network excitation A_ϵ, the quiescence period I_ϵ or the avalanche size s, we calculate the Kullback–Leibler (KL) divergence [28]:

$$D_{\rm dm}(X) \equiv D_{KL}(P_{\rm d}(X) \| P_{\rm m}(X)) = \sum_{A_\epsilon} P_{\rm d}(X) \ln \frac{P_{\rm d}(X)}{P_{\rm m}(X)}. \tag{33}$$

This is to be compared with the average KL divergence across subjects: $D_{\rm dd}(X) = \langle D_{KL}(P^i_{\rm d}(X) \| P^j_{\rm d}(X)) \rangle_{i,j}$, averaged across all pairs of MEG subjects indexed by i and j. The data-model divergence is very small and within the range of variability across subjects ($D_{\rm dm}(X) \lesssim D_{\rm dd}(X)$, see Table 1) for all distributions analyzed ($P(A_\epsilon)$, $P(I_\epsilon)$, and $P(s)$), suggesting that the model quantitatively reproduces the measured distributions to the degree that can be expected given natural variability in the data. Alternatively, we compared Eq. 33 with an average KL divergence across subjects calculated as $D_{\rm dd}(X) = \langle D_{KL}(P^i_{\rm d}(X) \| P_{\rm d}(X)) \rangle_i$, namely comparing each subject with the average distribution. Results were comparable to those shown in Table 1.

Distribution	KL Divergence among subjects (mean ± SD)	KL Divergence Data vs Model
$P(A_\varepsilon)$	0.0169 ± 0.0076	0.0092
$P(I_\varepsilon)$	0.0363 ± 0.0232	0.0107
$P(s)$	0.2823 ± 0.0662	0.1715

Supplementary Table 1 The adaptive Ising model that reproduces signal autocorrelation on an individual MEG sensor makes quantitative predictions about the distribution of network excitation [$P(A_\epsilon)$], the distribution of quiescence durations [$P(I_\epsilon)$], and the distribution of avalanche sizes [$P(s)$], collective quantities defined over the entire MEG sensor array. Average (± SD) Kullback-Leibler (KL) divergence across all pairs of subjects (second column) quantifies the biological variability in these distributions. The mismatch between model-predicted and data-derived (across-subject-average) distributions is also quantified by the KL divergence and reported in the third row (computed as in Eq (33)).

3 Supplementary Section-3. Supplementary figures

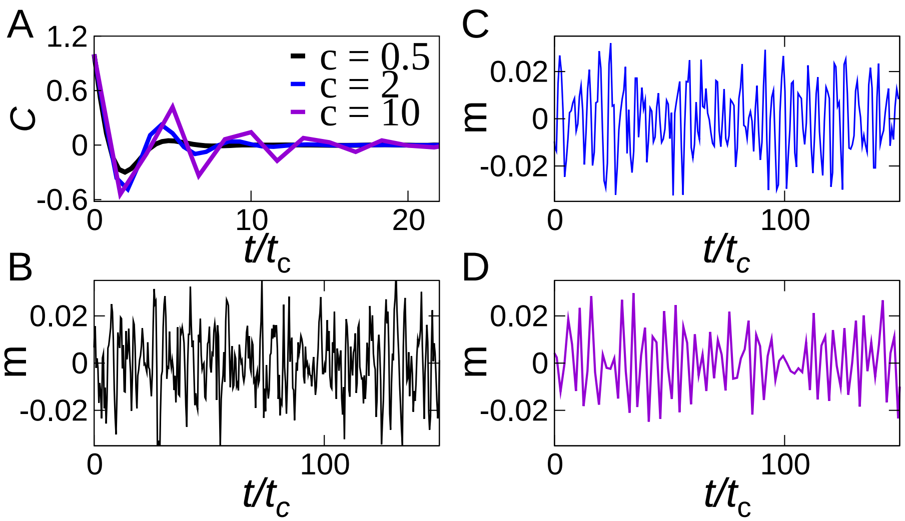

Supplementary Figure 7 Analysis of autocorrelation C and ongoing network activity m for $\beta = 0.5$ and different c values. Far from the critical point, the presence of a strong adaptive feedback may also produce short — C rapidly decays to zero — intermittent oscillation bursts. (A) Autocorrelation for different c values. (B) m for $c = 0.5$. (C) m for $c = 2$. (D) m for $c = 10$. t_c is the inferred autocorrelation time from exponential fit.

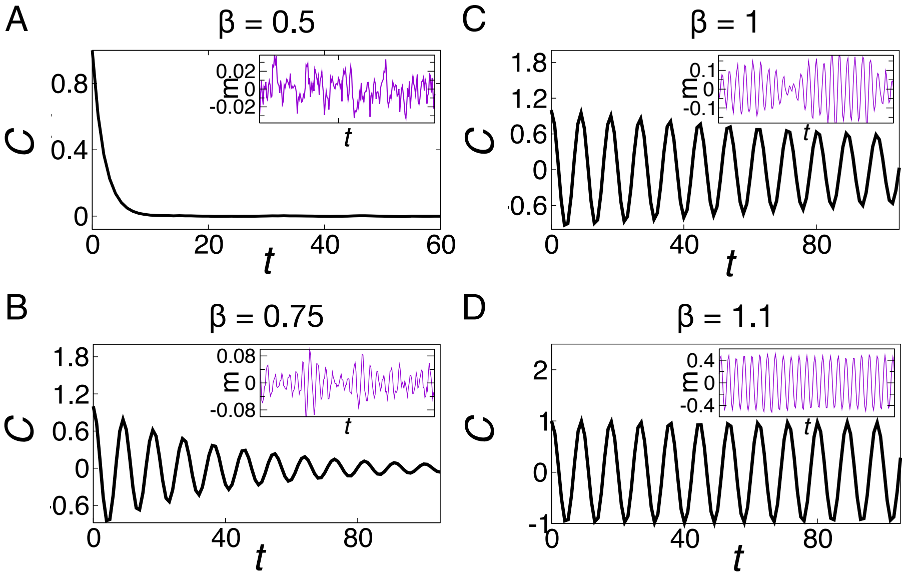

Supplementary Figure 8 Analysis of the autocorrelation C and ongoing network activity m for $c = 0.5$ and different values of the parameter β controlling proximity to the critical/bifurcation point β_c. In all cases the system is the resonant regime, and well above the transition line $c = c^*$ (see Fig. 1b). However, we observe that the system only develops consistent and structured oscillations for large enough β values, namely closer to the critical point $\beta = 1$. For $\beta > 1$, the system exhibits self-oscillations.

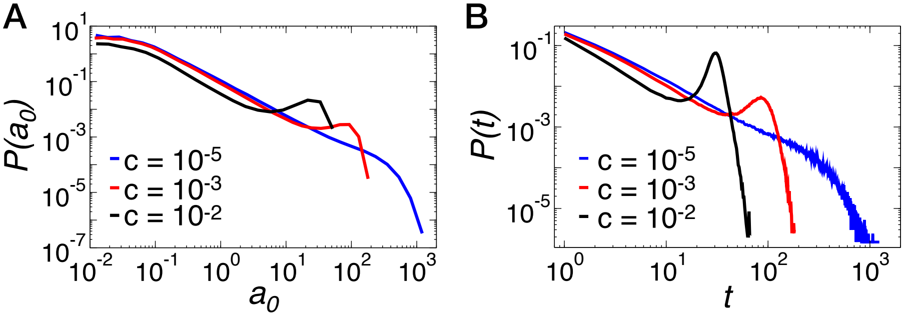

Supplementary Figure 9 Analysis of the reversal time and of the area under the curve for the network activity m. The reversal time t is defined as the time interval between consecutive zero-crossing events in the ongoing network activity m (Supplementary Figure 1). The quantity a_0 is the area under the curve between two zero-crossing events. (A) Distribution $P(a_0)$ of the quantity a_0 for the model at the critical point $\beta = 1$ for the different strengths c of the adaptive feedback. (B) Distribution $P(t)$ of the reversal time for $\beta = 1$ and different values of the parameter c.

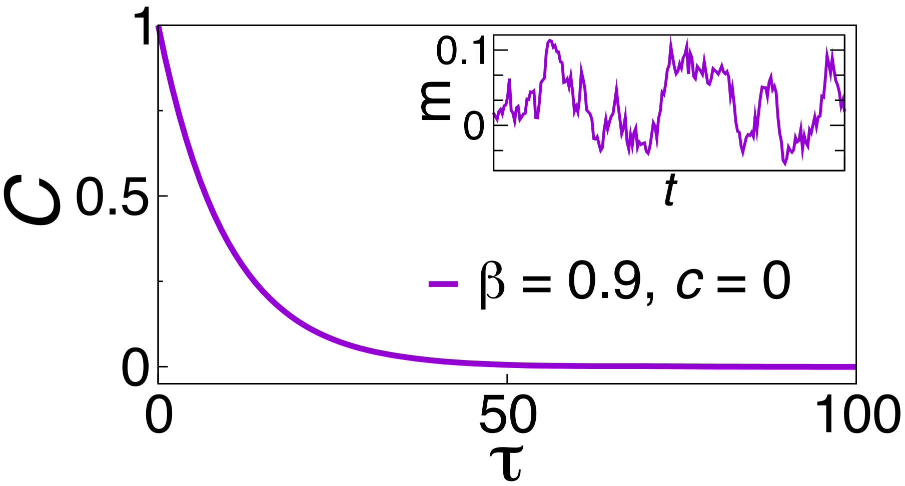

Supplementary Figure 10 The autocorrelation C and ongoing network activity m (inset) without adaptive feedback, i.e. $c = 0$. Even though β is close to the critical value $\beta = 1$, the network does not exhibit any oscillatory behavior.

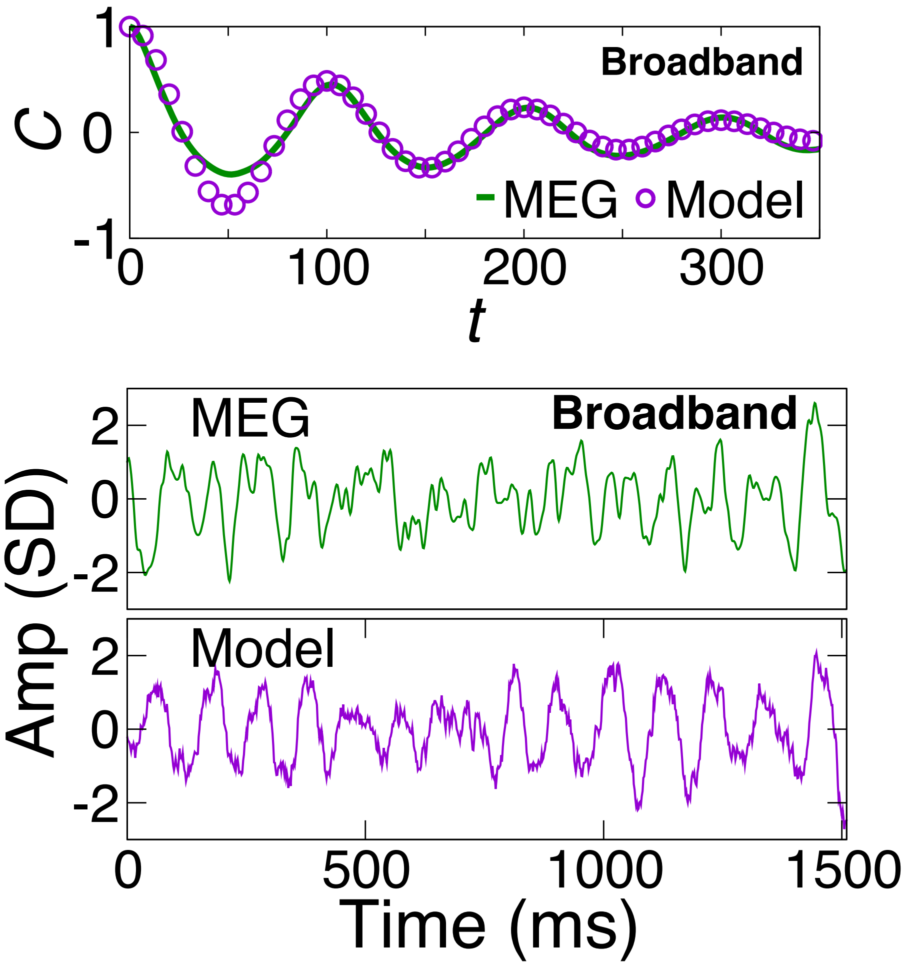

Supplementary Figure 11 Inference of network state from broadband signals. (Top panel) Broadband MEG signal autocorrelation and corresponding model autocorrelation for inferred parameters. (Bottom panel) The broadband signal from a single MEG sensor in the resting awake state is compared with the network activity m from model simulations with parameters $\beta = 0.99$ and $c = 0.01$ inferred from the single sensor signal.

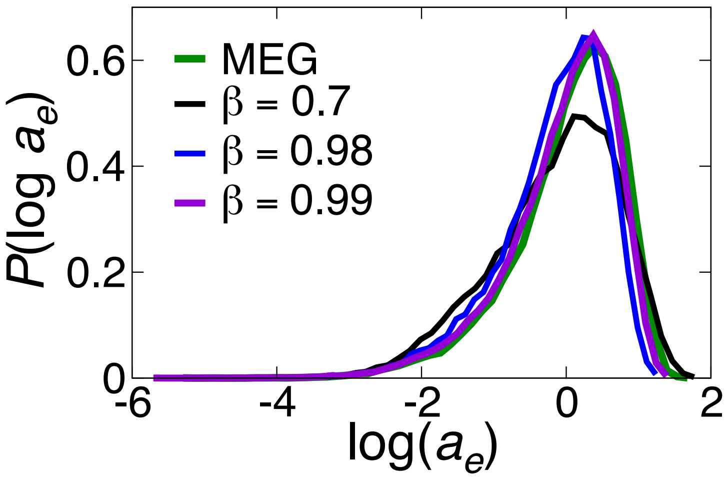

Supplementary Figure 12 Dependence of the distribution $P(\log a_e)$ on the value of β. Distributions $P(\log a_e)$ of the logarithm of the area under the curve a_e, with $e = 2.5$SD, for MEG data (green curves = average over sensors for one subject) and the model with different β values and $c = 0.5$.

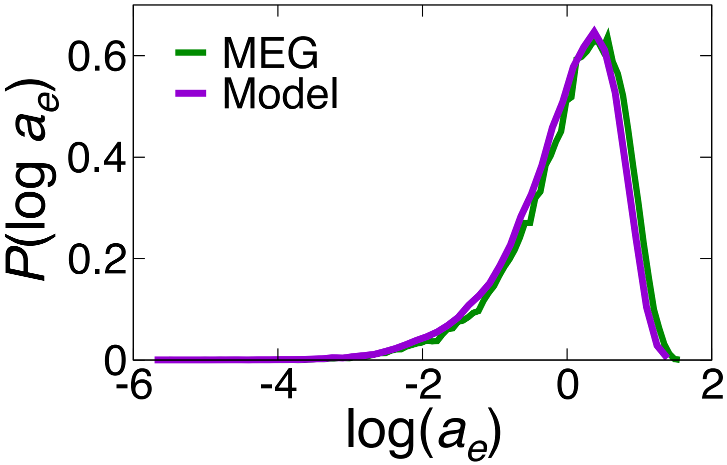

Supplementary Figure 13 Analysis of the distribution $P(\log a_e)$ of the logarithm of the area under the curve a_e for a single subject. Distributions $P(\log a_e)$ for a single for MEG data (green curves = average over sensors for one subject) and the model with corresponding inferred parameter values. The threshold value is $e = 2.5$SD (see Fig. 2).

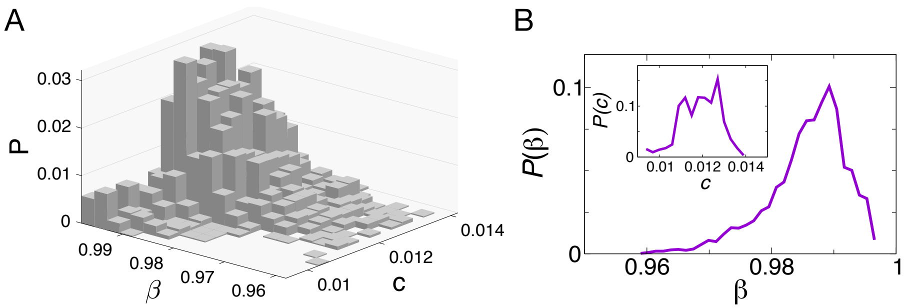

Supplementary Figure 14 Inferred parameter values across all subjects and sensors. (A) Joint probability of inferred values of the two model parameters from all MEG sensors (273) and subjects (14). (B) Marginal probability distributions of the inferred model parameters β (main panel) and c (inset).

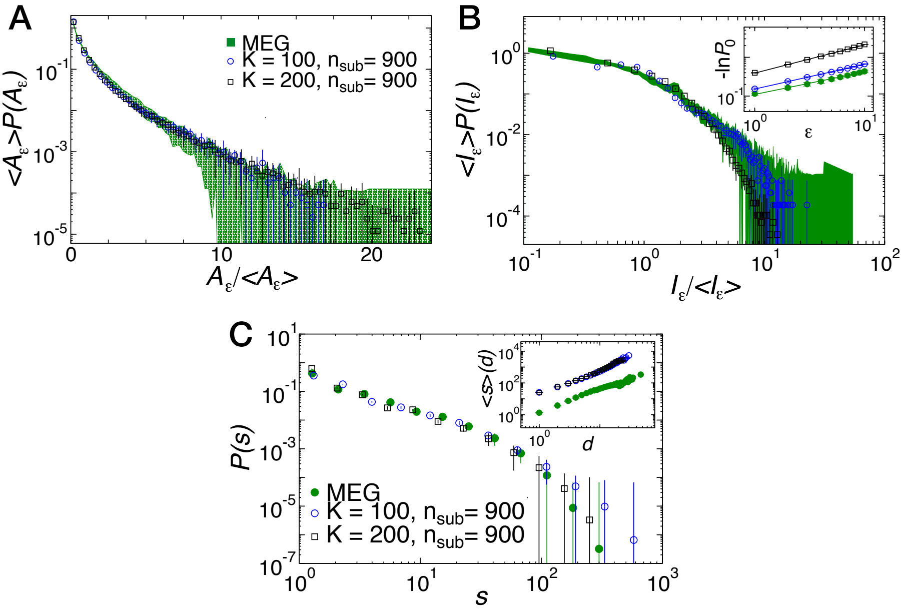

Supplementary Figure 15 Analysis of the distribution $P(A_\epsilon)$, $P(I_\epsilon)$, and $P(s)$ in model simulations for different numbers of subsystems K with a fixed number of neurons $n_{sub} = 900$. (A) The distribution of network excitation $P(A_\epsilon)$ ($\epsilon = \epsilon_2 = 2T$) does not depend on the number of equally sized subsystems $K = N/n_{sub}$ (green shaded area = mean $\pm$ SD; $n = 14$ MEG subjects). (B) The distribution of quiescence durations $P(I_\epsilon)$ ($\epsilon = \epsilon_2 = 2T$) show a weak dependence on the number of equally sized subsystems $K = N/n_{sub}$ for large I_ϵ (green shaded area = mean $\pm$ SD; $n = 14$ MEG subjects). Inset: Probability P_0 of finding a quiescent time bin scales approximately as $P_0 = \exp\left(-r_0\epsilon^{\beta_I}\right)$ with bin size ϵ; $\beta_I \simeq 0.6$ depends on the number of subsystems N/n_{sub}, and slightly increases from ≈ 0.6 for $K = 100$ (blue circles) to ≈ 0.7 for $K = 200$ (black squares). (C) The distribution of avalanche sizes $P(s)$ weakly depends on the number of equally sized subsystems K (green circles = mean $\pm$ SD; $n = 14$ MEG subjects). Inset: The relationship between average avalanche sizes and durations is independent of K. $P(s)$ distributions are calculated with $\epsilon = \epsilon_2 = 2T$. Error bars represent the standard deviation. All simulations were performed at $\beta = 0.99$, $c = 0.01$. For $K = 100$ ($K = 200$) the total number of neurons is $N = 9 \cdot 10^4$ ($N = 1.8 \cdot 10^5$).

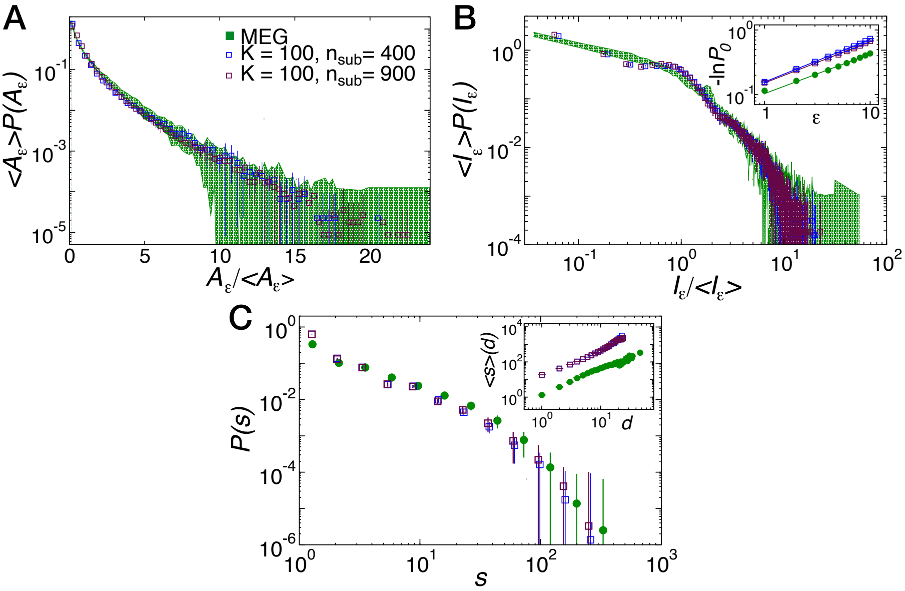

Supplementary Figure 16 Analysis of the distribution $P(A_\epsilon)$, $P(I_\epsilon)$, and $P(s)$ in model simulations for a fixed number of subsystems $K = 100$ and different subsystem size n_{sub}. (A) The distribution of network excitation $P(A_\epsilon)$ ($\epsilon = \epsilon_2 = 2T$) is robust to change in n_{sub} (shaded green area = mean ± SD; $n = 14$ MEG subjects). (B) The distribution of quiescence durations $P(I_\epsilon)$ ($\epsilon = \epsilon_2 = 2T$) does not depend on n_{sub} for fixed K (shaded green area = mean ± SD; $n = 14$ MEG subjects). Inset: Probability P_0 of finding a quiescent time bin scales approximately as $P_0 = \exp\left(-r_0\epsilon^{\beta_I}\right)$ with bin size ϵ; $\beta_I \simeq 0.6$ for both $n_{sub} = 400$ (blue squares) and $n_{sub} = 900$ (maroon squares). (C) The distribution of avalanche sizes $P(s)$ weakly depends on the subsystem size (green circles = mean ± SD; $n = 14$ MEG subjects). Inset: The relationship between average avalanche sizes and durations is independent of n_{sub}. $P(s)$ distributions are calculated with $\epsilon = \epsilon_4 = 4T$. Error bars represent the standard deviation. All simulations were performed at $\beta = 0.99$, $c = 0.01$.

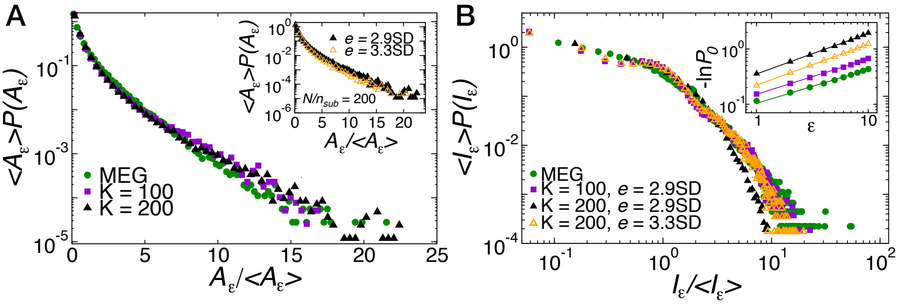

Supplementary Figure 17 Analysis of the distributions $P(A_\epsilon)$ and $P(I_\epsilon)$ in model simulations with different numbers of subsystems $K = N/n_{sub}$. (A) The distribution of network excitation $P(A_\epsilon)$ ($\epsilon = \epsilon_2 = 2T$) weakly depends on the number of subsystems $K = N/n_{sub}$. Inset: Distributions $P(A_\epsilon)$ for different values of the threshold e used to detect extreme events. The number of subsystems is fixed to $N/n_{sub} = 200$. (B) The distribution of quiescence durations $P(I_\epsilon)$ ($\epsilon = \epsilon_2 = 2T$) depends on the number of subsystems $K = N/n_{sub}$, particularly on the tail (black triangles up). For $K = 200$ a good agreement between data and model simulations is recovered when the threshold e is increased from 2.9SD (the value used for $K = 100$) to 3.3SD (orange triangles up). Inset: Probability P_0 of finding a quiescent time bin scales approximately as $P_0 = \exp\left(-r_0\epsilon^{\beta_I}\right)$ with bin size ϵ; $\beta_I \simeq 0.6$ depends on the number of subsystems K, and slightly increases from ≈ 0.6 for $K = 100$ (violet squares) to ≈ 0.7 for $K = 200$ (black triangles).

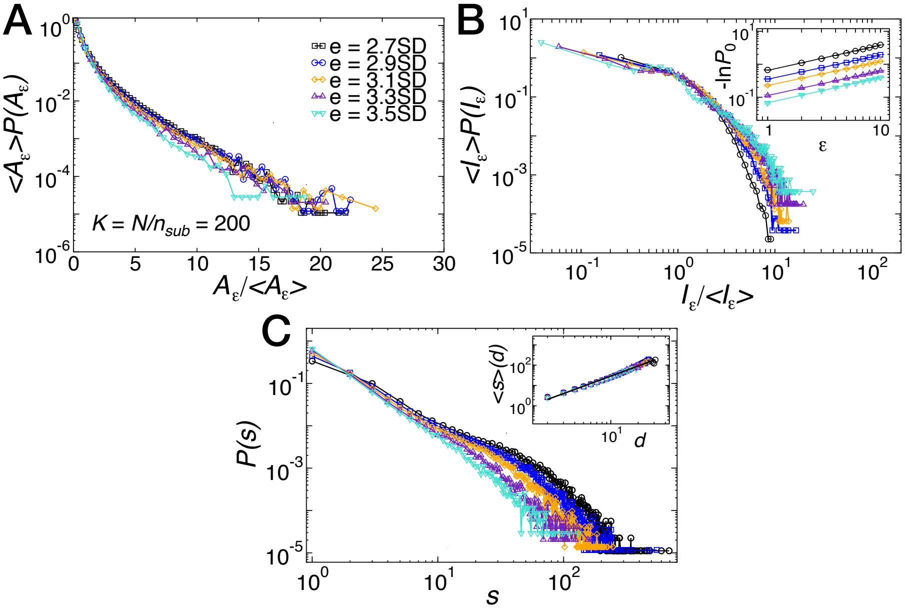

Supplementary Figure 18 Dependence of the distributions $P(A_\epsilon)$, $P(I_\epsilon)$, and $P(s)$ on the threshold e used to define extreme events and neuronal avalanches in model simulations. (A) For increasing e values, the probability of larger A_ϵ decreases while the probability of smaller A_ϵ decreases. However, the functional form of the distribution is stable. (B) For increasing e values, the probability of larger (smaller) I_ϵ increases (decreases), while the functional form of the distribution is stable. Inset: Probability P_0 of finding a quiescent time bin scales approximately as $P_0 = \exp\left(-r_0\epsilon^{\beta_I}\right)$ with bin size ϵ; the exponent β_I weakly depends on e, and slightly increases from ≈ 0.6 for $e = 2.7$ SD (black circles) to ≈ 0.7 for $e = 3.5$SD (turquoise triangles). (C) As in (A), increasing e leads to a decrease (increase) in the likelihood of observing large (small) avalanches. This effect particularly affects the tail of the distribution, while the scaling regime ($1 < s < 20$) is rather stable. Inset: The scaling relation between avalanche sizes and durations is independent of e, and follows a power law with an exponent $\zeta \simeq 1.5$. All distributions are calculated with $\epsilon = \epsilon_2 = 2T$. Model simulations: $N = 9 \cdot 10^4$ spins ($\beta = 0.99$, $c = 0.01$) and $K = 200$.

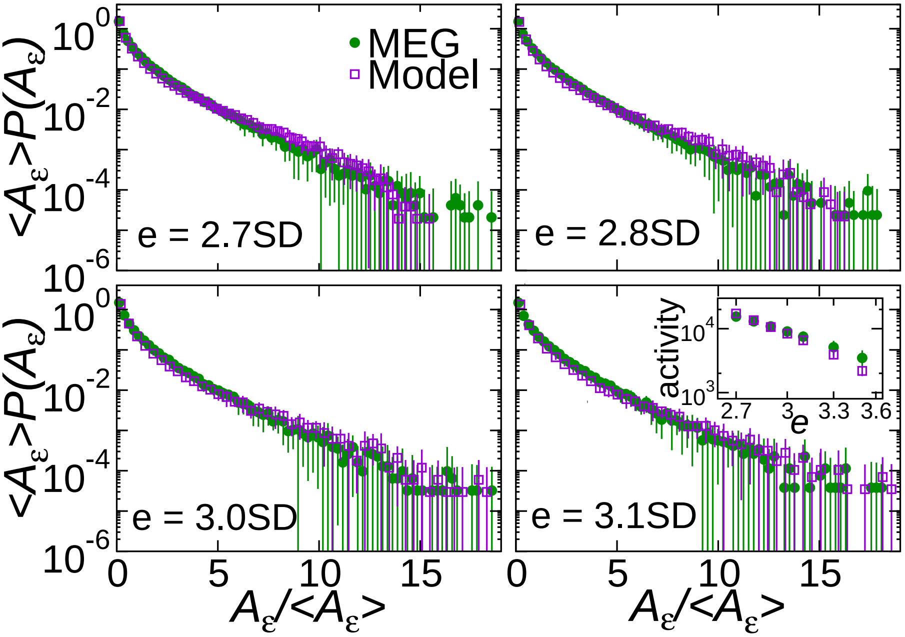

Supplementary Figure 19 Comparison of the distribution of network excitation $P(A_\epsilon)$ in MEG data and model simulations for different values of the threshold e. The distribution $P(A_\epsilon)$ changes with e in a similar way in both data and model simulations, and simulations reproduce empirical data within their range of variability in a range of e values around $e = 2.9SD$, the value used in the Fig. 3 (main text). Inset: Total number of extreme events as a function of the threshold e. This number decreases of about one order of magnitude when e increases from 2.7SD to 3.5SD. MEG distributions: mean ± SD; $n = 14$ subjects. Model distributions: simulations at baseline parameters ($\beta = 0.99$, $c = 0.01$) for a network of $N = 90000$ with $K = 100$. Errors bars represent the standard deviation. All distributions are calculated with $\epsilon = \epsilon_2 = 2T$.

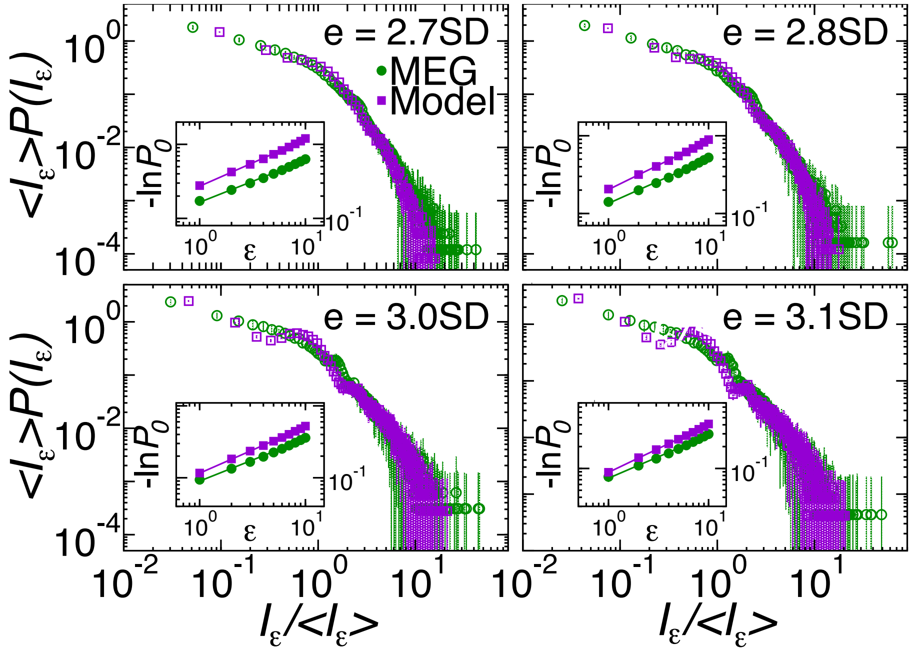

Supplementary Figure 20 Comparison of the distribution of quiescence durations $P(I_\epsilon)$ in MEG data and model simulations for different values of the threshold e. The distribution $P(I_\epsilon)$ changes with e in a similar way in both data and model simulations, and simulations reproduce empirical data within their range of variability over a range of e values around $e = 2.9SD$, the value used in Fig. 3 (main text). Insets: Probability P_0 of finding a quiescent time bin scales approximately as $P_0 = \exp\left(-r_0\epsilon^{\beta_I}\right)$ with bin size ϵ; the exponent β_I tend to increase for increasing e's in both data (from $\beta_I = 0.57 \pm 0.01$ at $e = 2.7$SD to $\beta_I = 0.59 \pm 0.01$ at $e = 3.1$SD; exponent $\pm$ error on the fit) and model (from $\beta_I = 0.63 \pm 0.01$ at $e = 2.7$SD to $\beta_I = 0.66 \pm 0.01$ at $e = 3.1$SD). MEG distributions: mean $\pm$ SD; $n = 14$ subjects. Model distributions: simulations at baseline parameters ($\beta = 0.99$, $c = 0.01$) for a network of $N = 90000$ with $K = 100$. Errors bars represent the standard deviation. All distributions are calculated with $\epsilon = \epsilon_2 = 2T$.

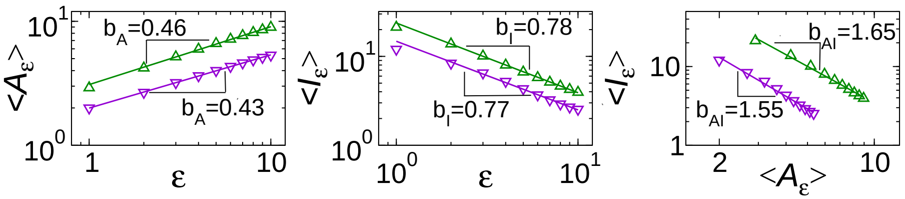

Supplementary Figure 21 Dependence of the average network excitation, $\langle A_\epsilon \rangle$, and average quiescence duration, $\langle I_\epsilon \rangle$, on the bin size ϵ. $\langle A_\epsilon \rangle$ and $\langle I_\epsilon \rangle$ scale as a power-law of the bin size ϵ, and are connected to each other by a power-law relationship. (Left panel) $\langle A_\epsilon \rangle$ scales with ϵ as $\langle A_\epsilon \rangle \sim \epsilon^{b_A}$, with similar exponents b_A in data and model simulations (Data: $b_A = 0.46 \pm 0.02$; Model: $b_A = 0.43 \pm 0.01$). (Middle panel) $\langle I_\epsilon \rangle$ scales with ϵ as $\langle I_\epsilon \rangle \sim \epsilon^{b_I}$, with similar exponents b_I in data and model simulations (Data: $b_I = 0.78 \pm 0.03$; Model: $b_A = 0.77 \pm 0.01$). (Right panel) $\langle I_\epsilon \rangle$ is connected to $\langle A_\epsilon \rangle$ by the relationship $\langle I_\epsilon \rangle \sim \langle A_\epsilon \rangle^{b_{AI}}$, with similar exponents b_{AI} in data and model simulations (Data: $b_{AI} = 1.65 \pm 0.12$; Model: $b_{AI} = 1.55 \pm 0.03$). Simulations are from a model with $N = 90000$ and $K = 100$. Extreme events are extracted using a threshold $e = 2.9$SD in both data and model simulations. For a given ϵ, the value of $\langle A_\epsilon \rangle$ and $\langle I_\epsilon \rangle$ can be controlled adjusting the threshold e. This implies that, beside the scaling exponents, it is possible to match the values of those quantities in the model by appropriately tuning e.

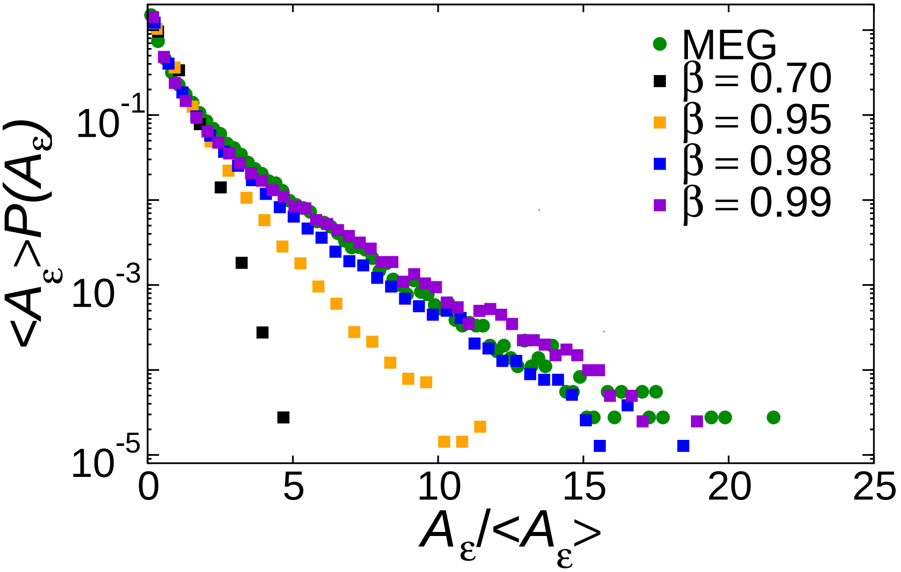

Supplementary Figure 22 Dependence of the distribution of network excitation $P(A_\epsilon)$ on β in model simulations. The model network is parceled in $K = 100$ disjoint subsystems, each including $n_{sub} = 1000$ spins ($N = 10^5$). In all cases the model is in the resonant regime ($c > c^*$). The network excitation A_ϵ is rescaled by the average network excitation $\langle A_\epsilon \rangle$ ($\epsilon = \epsilon_2 = 2T$, where T is the sampling interval, as in Fig. 3). $\beta = 0.99$ corresponds to the average β value inferred from MEG data. MEG data = average over subjects (green circles).

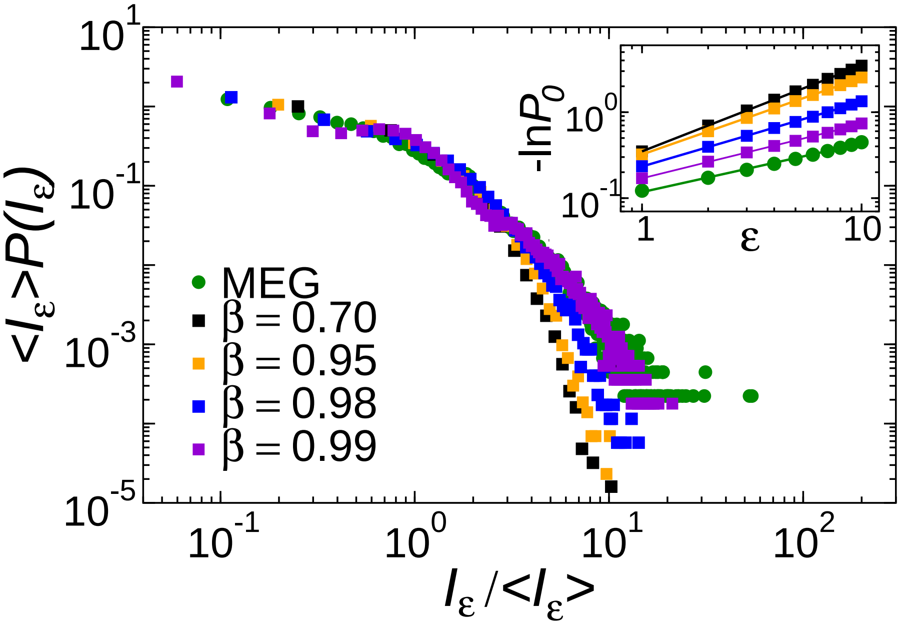

Supplementary Figure 23 Dependence of the distribution of quiescence durations $P(I_\epsilon)$ on β in model simulations. The model network is parceled in $K = 100$ disjoint subsystems, each including $n_{sub} = 1000$ spins ($N = 10^5$). In all cases the model is in the resonant regime ($c > c^*$). The quiescence duration I_ϵ is rescaled by the average quiescence duration $\langle I_\epsilon \rangle$ ($\epsilon = \epsilon_2 = 2T$, where T is the sampling interval, as in Fig. 3). $\beta = 0.99$ corresponds to the average β value inferred from MEG data. MEG data = average over subjects (green circles). Inset: Probability P_0 of quiescence periods as a function of ϵ for different β values. We notice that as we move away from the critical point $\beta_c = 1$, the probability tends to follow an exponential behavior, i.e. $P_0 \propto e^{-a\epsilon}$. On the other hand, for $\beta = 0.99$ we find $P_0 \propto e^{-a\epsilon^{\beta_I}}$, with $\beta_I = 0.6304 \pm 0.0046$, close to the value measured in MEG data (green circles) ($\beta_I = 0.5669 \pm 0.0117$)).

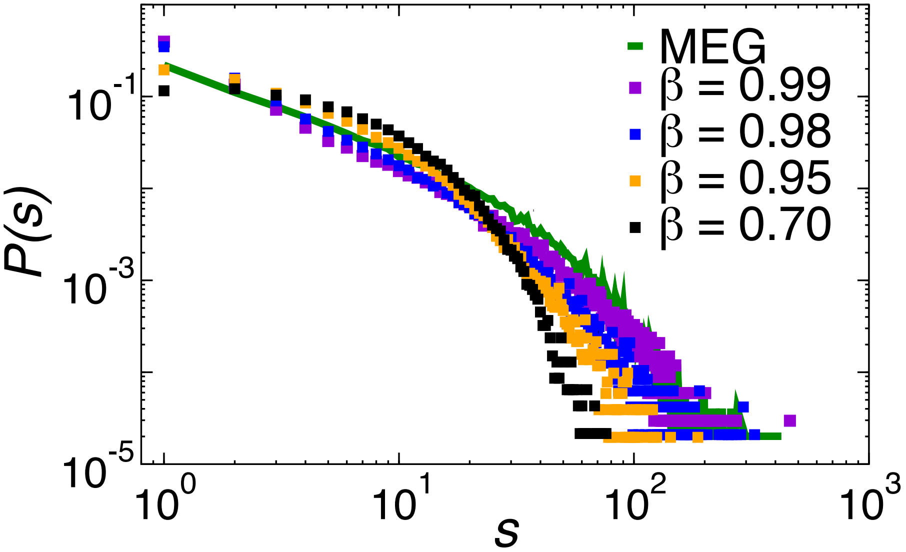

Supplementary Figure 24 Analysis of the distribution of avalanche sizes, $P(s)$, for the model simulated at different β values in the resonant regime ($c > c^*$). The model network is parceled in $K = 100$ disjoint subsystems, each including $n_{sub} = 1000$ spins ($N = 10^5$). Distributions are estimated using a threshold $e = 2.9$SD and bin size $\epsilon_4 = 4T$. Already for $\beta = 0.95$, a value slightly smaller than the baseline value 0.99, avalanche sizes from model simulations tend to follow an exponential distribution that is far from reproducing avalanche size distributions from MEG data.

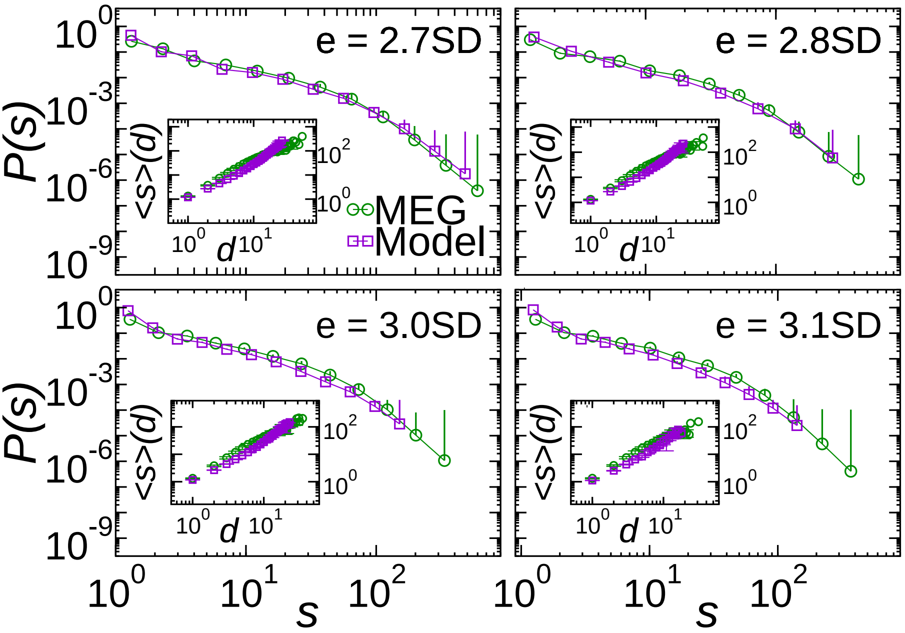

Supplementary Figure 25 Comparison of the distribution of avalanche sizes $P(s)$ in MEG data and model simulations at different values of the threshold e. The distribution $P(s)$ changes with e in a similar way in both data and model simulations, and simulations reproduce empirical avalanche size distributions within their range of variability over a range of e values around $e = 2.9$SD, which is used in Fig. 3 (main text). Insets: The relationship between the average avalanche size and the avalanche duration is robust and follows a power law with an exponent ζ that is roughly independent of e both in MEG data (from $\zeta = 1.28 \pm 0.03$ at $e = 2.7$SD, to $\zeta = 1.32 \pm 0.05$ at $e = 3.1$SD; exponent $\pm$ error on the fit) and model simulations (from $\zeta = 1.67 \pm 0.05$ at $e = 2.7$SD, to $\zeta = 1.58 \pm 0.05$ at $e = 3.1$SD). MEG distributions: mean $\pm$ SD ($n = 14$ subjects). Model distributions: simulations at baseline parameters ($\beta = 0.99$, $c = 0.01$) for a network of $N = 90000$ with $K = 100$. Errors bars represent the standard deviation.

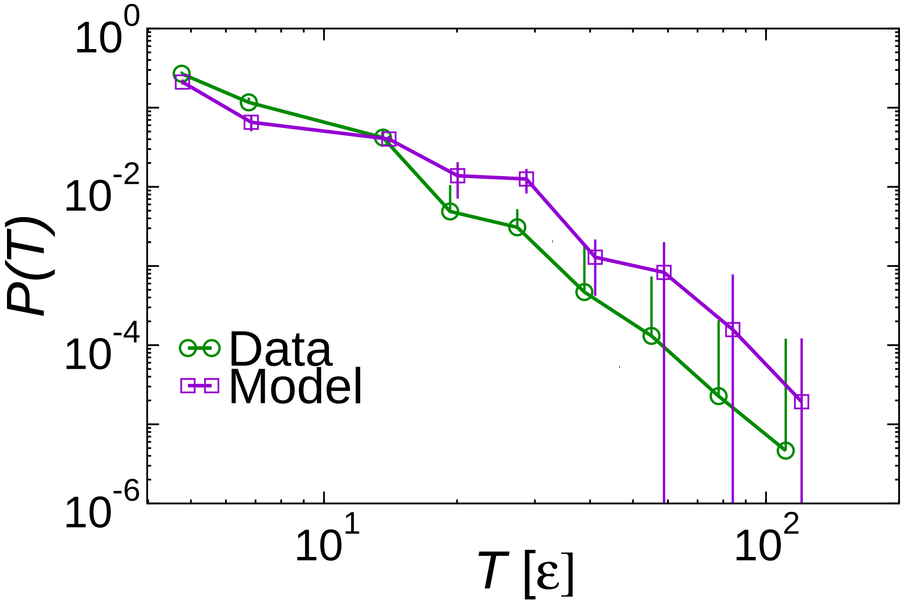

Supplementary Figure 26 Reproducing the distribution of neuronal avalanche durations in MEG resting state activity with a marginally subcritical adaptive Ising model. Distribution of avalanche durations, $P(T)$, for MEG data (green curve = mean ± SD; $n = 14$ subjects) and the model simulated at $\beta = 0.99$ and $c = 0.01$. Distributions are estimated using a threshold $e = 2.9$SD and bin size $\epsilon_4 = 4T$.

References

[1] Bonifazi, P., Goldin, M., Picardo, M.A., Jorquera, I., Cattani, A., Bianconi, G., Represa, A., Ben-Ari, Y., Cossart, R.: Gabaergic hub neurons orchestrate synchrony in developing hippocampal networks. Science **326**(5958), 1419–1424 (2009)

[2] Gardiner, C.: Stochastic Methods vol. 4. Springer, Berlin (2009)

[3] Seifert, U.: Stochastic thermodynamics, fluctuation theorems and molecular machines. Rep. Prog. Phys. **75**, 126001 (2012)

[4] Peliti, L.: On the work–hamiltonian connection in manipulated systems. J. Stat. Mech. **P05002** (2008)

[5] Lynn, C.W., Cornblath, E.J., Papadopoulos, L., Bertolero, M.A., Bassett, D.S.: Broken detailed balance and entropy production in the human brain. Proc Natl Acad Sci USA **118**(47), 1–7 (2021)

[6] Buhmann, J., Schulten, K.: Noise-driven temporal association in neural networks. EPL **4**, 1205 (1987)

[7] Buzsaki, G.: Rhythms of the Brain. Oxford University Press, ??? (2006)

[8] Arenas, A., Díaz-Guilera, A., Kurths, J., Moreno, Y., Zhou, C.: Synchronization in complex networks. Physics reports **469**(3), 93–153 (2008)

[9] Adiwes international series in physics. In: Andronov, A.A., Vitt, A.A., Khaikin, S.E. (eds.) Theory of Oscillators. Adiwes International Series in Physics. Pergamon (1966)

[10] Peron, T., de Resende, B.M.F., Mata, A.S., Rodrigues, F.A., Moreno, Y.: Onset of synchronization of kuramoto oscillators in scale-free networks. Physical Review E **100**(4), 042302 (2019)

[11] Onsager, L.: Crystal statistics. i. a two-dimensional model with an order-disorder transition. Physical Review **65**(3-4), 117 (1944)

[12] Baxter, R.J.: Exactly Solved Models in Statistical Mechanics. Academic Press, ??? (1982)

[13] Dorogovtsev, S.N., Goltsev, A.V., Mendes, J.F.: Critical phenomena in complex networks. Reviews of Modern Physics **80**(4), 1275 (2008)

[14] Newman, M.: Networks. Oxford University Press, ??? (2018)

[15] Leone, M., Vázquez, A., Vespignani, A., Zecchina, R.: Ferromagnetic

ordering in graphs with arbitrary degree distribution. The European Physical Journal B-Condensed Matter and Complex Systems **28**(2), 191–197 (2002)

[16] Carvalho, T.T.A., Fontenele, A.J., Girardi-Schappo, M., Feliciano, T., Aguiar, L.A.A., Silva, T.P.L., de Vasconcelos, N.A.P., Carelli, P.V., Copelli, M.: Subsampled directed-percolation models explain scaling relations experimentally observed in the brain. Front. Neural Circuits **14**, 576727 (2021). https://doi.org/10.3389/fncir.2020.576727

[17] Fontenele, A.J., de Vasconcelos, N.A.P., Feliciano, T., Aguiar, L.A.A., Soares-Cunha, C., Coimbra, B., Porta, L.D., Ribeiro, S., Rodrigues, A.J., Sousa, N., Carelli, P.V., Copelli, M.: Criticality between cortical states. Phys. Rev. Lett. **122**(20), 208101 (2019)

[18] Peng, C.-K., Buldyrev, S.V., Havlin, S., Simons, M., Stanley, H.E., Goldberger, A.L.: Mosaic organization of dna nucleotides. Physical review E **49**(2), 1685 (1994)

[19] Linkenkaer-Hansen, K., Nikouline, V.V., Palva, J.M., IImoniemi, R.J.: Long-range temporal correlations and scaling behavior in human brain oscillations. Journal of Neuroscience **21**(4), 1370–1377 (2001)

[20] Hardstone, R., Poil, S.-S., Schiavone, G., Jansen, R., Mansvelder, V.V.N.H.D., Linkenkaer-Hansen, K.: Detrended fluctuation analysis: a scale-free view on neuronal oscillations. Front. in Physiol. **3**, 450 (2012)

[21] Shriki, O., Alstott, J., Carver, F., Holroyd, T., Hanson, R.N.A., Smith, M.L., Coppola, R., Bullmore, E., Plenz, D.: Neuronal avalanches in the resting meg of the human brain. J. Neurosci. **33**(16), 7079–7090 (2013)

[22] Lombardi, F., Shriki, O., Herrmann, H.J., de Arcangelis, L.: Long-range temporal correlations in the broadband resting state activity of the human brain revealed by neuronal avalanches. Neurocomputing **461**, 657–666 (2021)

[23] Janke, W., Johnston, D.A., Kenna, R.: Information geometry and phase transitions. Physica A **336**(1–2), 181–186 (2004)

[24] Pausch, J., Garcia-Millan, R., Pruessner, G.: Time-dependent branching processes: a model of oscillating neuronal avalanches. Sci. Rep. **10**, 13678 (2020)

[25] Ferretti, F., Chardès, V., Mora, T., Walczak, A.M., Giardina, I.: Building general langevin models from discrete datasets. Physical review X **10**, 031018 (2020)

[26] Brückner, D.B., Ronceray, P., Broedersz, C.P.: Inferring the dynamics of underdamped stochastic systems. Phys. Rev. Lett **125**, 058103 (2020)

[27] Theiler, J., Eubank, S., Longtin, A., Galdrikian, B., Farmer, J.D.: Testing nonlinearity in time series: the method of surrogate data. Physica D **58**, 77–94 (1992)

[28] Kullback, S., Leibler, R.A.: On information and sufficiency. Ann. Math. Statist. **22**, 79–86 (1951)
