# Supplementary material for: Statistical modeling of adaptive neural networks explains co-existence of avalanches and oscillations in resting human brain
Source: Nat Comput Sci. 2023 Mar 20;3(3):254–63. doi: 10.1038/s43588-023-00410-9 (PMC10766559; doi:10.1038/s43588-023-00410-9)
Supplement: Supplementary file 2 — Reporting Summary [file 43588_2023_410_MOESM2_ESM.pdf]

nature portfolio

Corresponding author(s): Fabrizio Lombardi

Last updated by author(s): Dec 26, 2022

Reporting Summary

Nature Portfolio wishes to improve the reproducibility of the work that we publish. This form provides structure for consistency and transparency in reporting. For further information on Nature Portfolio policies, see our Editorial Policies and the Editorial Policy Checklist.

Statistics

For all statistical analyses, confirm that the following items are present in the figure legend, table legend, main text, or Methods section.

n/a	Confirmed	
☐	☒	The exact sample size (*n*) for each experimental group/condition, given as a discrete number and unit of measurement
☒	☐	A statement on whether measurements were taken from distinct samples or whether the same sample was measured repeatedly
☐	☒	The statistical test(s) used AND whether they are one- or two-sided<br>*Only common tests should be described solely by name; describe more complex techniques in the Methods section.*
☒	☐	A description of all covariates tested
☒	☐	A description of any assumptions or corrections, such as tests of normality and adjustment for multiple comparisons
☐	☒	A full description of the statistical parameters including central tendency (e.g. means) or other basic estimates (e.g. regression coefficient) AND variation (e.g. standard deviation) or associated estimates of uncertainty (e.g. confidence intervals)
☐	☒	For null hypothesis testing, the test statistic (e.g. *F*, *t*, *r*) with confidence intervals, effect sizes, degrees of freedom and *P* value noted<br>*Give P values as exact values whenever suitable.*
☒	☐	For Bayesian analysis, information on the choice of priors and Markov chain Monte Carlo settings
☒	☐	For hierarchical and complex designs, identification of the appropriate level for tests and full reporting of outcomes
☒	☐	Estimates of effect sizes (e.g. Cohen's *d*, Pearson's *r*), indicating how they were calculated

Our web collection on statistics for biologists contains articles on many of the points above.

Software and code

Policy information about availability of computer code

Data collection	The data was recorded using a CTF MEG system (CTF Systems). Data preprocessing was performed using the Field-Trip toolbox (version 2011-09) in MATLAB 2011a (Mathworks)
Data analysis	Data analysis was performed in MATLAB 2020b.

For manuscripts utilizing custom algorithms or software that are central to the research but not yet described in published literature, software must be made available to editors and reviewers. We strongly encourage code deposition in a community repository (e.g. GitHub). See the Nature Portfolio guidelines for submitting code & software for further information.

Data

Policy information about availability of data

All manuscripts must include a data availability statement. This statement should provide the following information, where applicable:

- Accession codes, unique identifiers, or web links for publicly available datasets
- A description of any restrictions on data availability
- For clinical datasets or third party data, please ensure that the statement adheres to our policy

The data analyzed in this study was collected at the MEG facility of the NIH. Data belongs to NIH and is available upon request. We have permission to use it and share it upon request. We do not have permission to upload the data into a public repository.

Human research participants

Policy information about studies involving human research participants and Sex and Gender in Research.

Reporting on sex and gender	Sex and gender were not relevant for the present study. We did not perform sex/gender based analyses. Although this information was collected for the original study (Shriki et al, J. Neurosci. 33 (16) : 7079 -7090, 2013; see Population characteristics), it was not accessible to the Authors and was not relevant for the selection of the 14 subjects used for the present study. Because we were interested in the connection between alpha oscillations and brain criticality, subject selection was solely based on the percentage of spectral power in the alpha band (8-13 Hz).
Population characteristics	The NIH facility recorded activity from 104 subjects (38 males and 66 females; age, 31.8 ± 11.8) for 4 min at rest with eyes closed (see Shriki et al, J. Neurosci. 33 (16) : 7079 -7090, 2013 for details).
Recruitment	Participants were healthy and had no history of neurological or psychiatric diseases.
Ethics oversight	NIH

Note that full information on the approval of the study protocol must also be provided in the manuscript.

Field-specific reporting

Please select the one below that is the best fit for your research. If you are not sure, read the appropriate sections before making your selection.

☒ Life sciences ☐ Behavioural & social sciences ☐ Ecological, evolutionary & environmental sciences

For a reference copy of the document with all sections, see nature.com/documents/nr-reporting-summary-flat.pdf

Life sciences study design

All studies must disclose on these points even when the disclosure is negative.

Sample size	The data analyzed here were selected from a set of MEG recordings for a previously published study (Shriki et al, J. Neurosci. 33 (16) : 7079 -7090, 2013), where further details can be found. For the present analyses we used the subjects (n = 14) showing the highest percentage of spectral power in the alpha band (8-13 Hz) (the dominant brain rhythm during resting wake). Similar results were obtained for randomly selected subjects. Results are very consistent across subjects, and increasing the sample size does not significantly influence the results. The number of selected subjects is thus adequate to provides robust results.
Data exclusions	In Shriki et al, J. Neurosci. 33 (16) : 7079 -7090, 2013, data from 104 participants recorded in the NIHM MEG core facility were considered. Because we were interested in the connection between alpha oscillations and brain criticality, for the present analyses we used 14 subjects showing high percentage of spectral power in the alpha band (8-13 Hz).
Replication	Experimental findings were replicated in a different MEG facility at the Medical Research Council Cognition and Brain Sciences Unit in Cambridge (see Shriki et al, J. Neurosci. 33 (16): 7079 -7090, 2013 for details). Data analyzed in the current study were previously used in Shriki et al, J. Neurosci. 33 (16) : 7079 -7090, 2013. Experimental findings were replicated once and presented in Shriki et al. J. Neurosci. 33 (16) : 7079 -7090, 2013.
Randomization	We analyzed data recorded from a single group of healthy subjects. Only participants with no history of neurological or psychiatric diseases were selected for this study. Results were consistent across subjects. Thus randomization was not relevant for the present study.
Blinding	Only brain activity recorded from healthy subjects was analyzed in this study. The data analyzed here were selected from a set of MEG recordings for a previously published study (Shriki et al, J. Neurosci. 33 (16) : 7079-7090, 2013). Because we were interested in the connection between alpha oscillations and brain criticality, we used 14 subjects showing high percentage of spectral power in the alpha band (8-13 Hz). Similar results were obtained for randomly selected subjects.

Reporting for specific materials, systems and methods

We require information from authors about some types of materials, experimental systems and methods used in many studies. Here, indicate whether each material, system or method listed is relevant to your study. If you are not sure if a list item applies to your research, read the appropriate section before selecting a response.

Materials & experimental systems

n/a	Involved in the study
☒	☐ Antibodies
☒	☐ Eukaryotic cell lines
☒	☐ Palaeontology and archaeology
☒	☐ Animals and other organisms
☒	☐ Clinical data
☒	☐ Dual use research of concern

Methods

n/a	Involved in the study
☒	☐ ChIP-seq
☒	☐ Flow cytometry
☒	☐ MRI-based neuroimaging
