# Supplementary material for: Statistical modeling of adaptive neural networks explains co-existence of avalanches and oscillations in resting human brain
Source: Nat Comput Sci. 2023 Mar 20;3(3):254–63. doi: 10.1038/s43588-023-00410-9 (PMC10766559; doi:10.1038/s43588-023-00410-9)
Supplement: Supplementary file 4 — Source Data for Supplementary Figs. 1–26. [file 43588_2023_410_MOESM4_ESM.zip › Supplementary_source_data_excel/README.rtf]

File name structure for main panels:fig_figure-number-panel_curve-legendExample: fig_S7a_c_0.5 contains data for the curve corresponding to c = 0.5 in the Supplementary Figure 7, panel aFile name structure for insets:fig_figure-number-panel_inset_curve-legendExample: fig_S15b_inset_meg contains data for the curve corresponding to MEG data in the inset of the Supplementary Figure 15, panel b.
