# Supplementary material for: Statistical modeling of adaptive neural networks explains co-existence of avalanches and oscillations in resting human brain
Source: Nat Comput Sci. 2023 Mar 20;3(3):254–63. doi: 10.1038/s43588-023-00410-9 (PMC10766559; doi:10.1038/s43588-023-00410-9)
Supplement: Supplementary file 5 — Statistical Source Data. [file 43588_2023_410_MOESM5_ESM.zip › fig_1_excel/README.rtf]

File name structure for main panels:fig_figure-number-panel_curve-legendExample: fig_1c_beta_0.7 contains data for the curve corresponding to beta = 0.7 in Fig. 1c.File name structure for insets:fig_figure-number-panel_inset_curve-legendExample: fig_1d_inset_P(t) contains data for the curve corresponding to P(t) in the inset of Fig. 1d.
